# Is nonoperative treatment of pediatric type I open fractures safe and effective?

**DOI:** 10.1007/s11832-014-0616-x

**Published:** 2014-11-06

**Authors:** Ahmed A. Bazzi, Jaysson T. Brooks, Amit Jain, Michael C. Ain, John E. Tis, Paul D. Sponseller

**Affiliations:** 1 Department of Orthopaedic Surgery, The Johns Hopkins University, 601 N. Caroline Street, Baltimore, MD 21287 USA; 2 C/o Rachel Box, Department of Orthopaedic Surgery, The Johns Hopkins University/Johns Hopkins Bayview Medical Center, 4940 Eastern Ave., #A665, Baltimore, MD 21224-2780 USA

**Keywords:** Pediatric open fracture, Type I open fracture, Nonoperative management of open fracture, Pediatric tibia fracture, Pediatric forearm fracture

## Abstract

**Purpose:**

There is limited literature on nonoperative treatment of open type I pediatric fractures. Our purpose was to evaluate the rate of infection in pediatric patients with type I open fractures treated nonoperatively at our institution without admission from the emergency department (ED).

**Methods:**

We performed a retrospective chart review of all patients who sustained a type I open fracture of the forearm or tibia from 2000 through 2013. Forty patients fit the inclusion criteria: <18 years old with type I open fracture treated nonoperatively with irrigation and debridement, followed by closed reduction and casting of the fracture under conscious sedation in the ED. All patients were discharged home. The primary outcome was presence of infection. Secondary outcomes included occurrence of a delayed union, time to union, complications, and residual angulation.

**Results:**

There were no reported or documented infections. There was one case of a retained foreign body (<1 cm) in a mid-diaphyseal forearm fracture, which was removed in clinic at 4 weeks after the patient developed a granuloma with no infectious sequela. There was one case of a delayed union; all patients eventually had complete bony union. There was minimal residual angulation in both upper and lower extremities at last follow-up.

**Conclusions:**

Nonoperative treatment of type I open fractures in pediatric patients can be performed safely with little risk of infection. This preliminary evidence may serve as a foundation for future prospective studies.

## Introduction

The current standard of care for an open fracture is formal irrigation and debridement in an operative setting because of the risk of contamination and infection. The rationale behind washing out the wounds of open fractures is that it cleanses the area of bacteria, with the intention of decreasing the incidence of osteomyelitis, nonunion, and malunion.

**Fig. 1:**
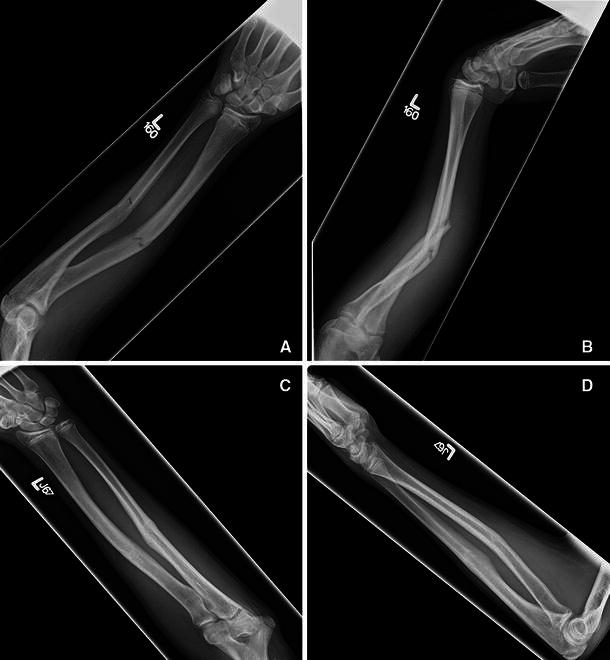
Radiographs of a 14-year-old boy with a type I open forearm fracture. **a**, **b** Postinjury anteroposterior and lateral radiographs at the time of evaluation in the emergency department. **c**, **d** Anteroposterior and lateral radiographs at 3-month follow-up showing complete bony union after nonoperative management

Recent literature has confirmed that the rate of wound infections and overall outcomes closely follows the open fracture type [1]. This classification of open fractures groups the lesions on the basis of the size of the wound and degree of contamination. Type I open fractures are those with a wound size ≤1 cm that have no evident contamination (Fig. 1a, b).

The treatment of open fractures in the adult population is well agreed upon as requiring irrigation and debridement in the operating room. In pediatrics, there is agreement only about the management of type II and III open fractures. The treatment of type I open fractures still results in considerable debate among pediatric orthopedic surgeons across the USA. Unfortunately, very little research exists on the nonoperative management of type I open forearm and tibial shaft fractures in children. To our knowledge, there are only two studies that evaluate the outcomes of nonoperative treatment of type I open fractures in pediatric patients [2, 3], but in both of those studies, patients were admitted for some period of time. In the pediatric orthopedic community, growing interest in nonoperative treatment of these injuries led to a 2012 survey by Wetzel et al. [4]. They found that although only 31 % of the 177 responding surgeons chose nonoperative treatment in an emergency department (ED) setting, 90 % indicated they would be open to changing their practice if level I evidence existed, and 122 (69 %) expressed interest in participating in a prospective, randomized trial on pediatric type I open fracture management.

Type I open fractures in pediatric patients have been treated nonoperatively for a number of years at our institution. Our purpose was to evaluate the rate of infection in pediatric patients with type I open fractures treated nonoperatively at our institution without admission from the ED.

## Methods

After obtaining approval by the institutional review board, we performed a retrospective search of our pediatric patient database for all those with open fractures (as defined by the International Classification of Diseases, 9th revision, Clinical Modification [5] codes 8249, 81313, 81318, 81333, 81354, 82310, 82312, 82330, or 82332) treated at our institution from 2000 through 2013. The records of those 302 patients were then reviewed to identify which ones met the inclusion criteria: (1) a diagnosis of a type I open fracture of the forearm or tibia, (2) age <18 years, and (3) fracture treatment received in an ED setting. Of the 302 patients, 262 were excluded because they had: a fracture that required initial operative treatment, a ballistic fracture, or a concomitant neurological or vascular injury. The 40 patients meeting the inclusion criteria formed our study group. All of these patients were discharged home after initial treatment in the ED.

Of the 40 patients, 35 had complete follow-up documentation in our electronic medical records, with an average follow-up time of 4.2 ± 8 months. Five patients were unable to return to clinic after their initial ED visit and had their casts removed at other institutions. Of those five, the parents of four patients were contacted by phone to inquire about any occurrence of an infection. The fifth patient followed up with her pediatrician at our institution, and clinical records were reviewed for infection.

Data collected included demographics such as age and sex, date of last follow-up, location of the fracture (distal, mid, or proximal forearm or tibia), antibiotic choice and duration, and mechanism of injury. The primary outcome was the presence of an infection (deep or superficial). Secondary outcomes included evidence of bony union (clinically and radiographically), presence of delayed union, complications, and any residual angulation observed. Most of the fractures in our 27 boys and 13 girls were both-bone forearm fractures, and the most common location for these fractures was the mid-forearm (Table 1). The mechanisms of injury were falls in 25 (63 %), moving cars in 12 (30 %), blunt injury in 2 (5 %), and motorcycle in 1 (2 %).

**Table 1:** Patient and fracture characteristics

Characteristics	*N* (%)
Sex	
Male	27 (67)
Female	13 (33)
Mean age, range (years)	8.6 (4–16)
Fracture type	
Radius-ulna	19 (48)
Monteggia	3 (7)
Ulna	2 (5)
Radius	2 (5)
Tibia-fibula	8 (20)
Tibia	6 (15)
Fracture location	
Proximal forearm	3 (8)
Mid-forearm	13 (32)
Distal forearm	11 (27)
Proximal leg	0
Mid-leg	6 (15)
Distal leg	7 (18)

Thirty-six patients (90 %) received a dose of intravenous antibiotics in the ED, and 28 patients (70 %) were discharged home with additional oral antibiotics. The mean oral antibiotic regimen was 7.7 days (range 3–14). The choice of the prescribed antibiotic was the treating ED physician’s preference: cephalexin in 25 patients (63 %) and clindamycin (because of penicillin allergies) in three patients (7 %).

Per our institution’s protocol for pediatric patients with type I open fractures, we started each patient on intravenous antibiotics while in the ED. Next, without any extension of the open fracture wound, a bedside irrigation was performed with saline or povidone-iodine mixed with saline, and debridement of any superficial foreign debris was also performed using gauze moistened with saline or the resident’s gloved finger as he or she continued to irrigate. The traumatic wound was then left open and covered with Xeroform (Covidien, Mansfield, MA) or nonadherent dressings. Subsequently, closed reduction of the fracture with cast application was performed, all under conscious sedation provided by the ED staff. An orthopedic resident performed the wound irrigation, fracture reduction, and casting of each patient with an open fracture. As mentioned previously, many patients were excluded secondary to operative treatment of their type I open fracture. Between 2000 and 2008, many type I open fractures were managed operatively at this institution based on the individual treatment preferences of the pediatric orthopedic attendings present at that time. In contrast to the authors’ method of nonoperative management, fractures treated operatively involved extension of the wound area for better exposure of the fracture site, debridement using curettes or rongeurs, and irrigation using a pulse lavage or cysto tubing.

## Results

There was no evidence of infection or cosmetic defect in any of the 35 patients who returned to the clinic. The parents of the four patients contacted by telephone reported that their children’s wound was well healed, that they did not require further care or treatment for an infection, and that there was no residual pain or deformity, according to their treating physicians. For the one patient who followed up with her pediatrician in our institution, there was no documented evidence of infection at the open fracture site.

The mean time to union was 92 days (range 30–180) for forearm fractures and 82 days (range 60–120) for tibia fractures. There was one case of a delayed union in a 4-year-old girl who sustained a type I open distal radius and ulna fracture after falling off monkey bars. Various options, including the use of a bone stimulator or surgery, were discussed with the parents, but they decided to continue with nonoperative management. She went on to complete bony union 7 months after her initial fracture and after 5 months of additional immobilization with a short arm fracture brace. All 35 patients with fractures who returned to our clinic for follow-up showed complete bony union, clinically and radiographically, by the last clinic visit (Fig. 1c, d). The mean time that patients spent in the emergency room from arrival to discharge was 7.7 ± 3.8 h.

There was one complication. A 6-year-old girl with a mid-diaphyseal both-bone forearm fracture was noted at her 4-week clinical examination to have retained a foreign body (<1 cm) that was walled off within a granuloma. It was removed in the clinic with no evidence of purulent drainage and no infectious sequelae.

Residual angulation was evaluated for the 35 patients who had complete radiographic data, revealing <10° overall angulation at the latest follow-up (Table 2).

**Table 2:** Residual angulation

Residual angulation	Mean measurement (° ± SD)
Radius	
Anteroposterior	6 ± 7
Lateral	5 ± 5
Ulna	
Anteroposterior	5 ± 4
Lateral	5 ± 4
Tibia	
Anteroposterior	3 ± 5
Lateral	4 ± 4

## Discussion

Prompt surgical debridement is considered a mainstay for care of open fractures. Recent studies, however, have challenged this concept, proving that time to washout is not an independent risk factor for wound infections in open fractures [6, 7]. Multiple studies have shown that the timing, choice, and duration of antibiotics are important factors in the treatment of open fractures [7–9]. The benefits of any open surgical debridement of the bone under these minor skin violations of type I fractures, by contrast, have not been shown. The wounds of many type I open fractures occurring in the extremities are often small poke holes; thus, further exposure of these types of fractures in the operating room would likely lead to more periosteal stripping and devascularization. Although treating these type I open fractures nonoperatively cannot be proven superior given the current study design, it is the senior author’s opinion that, compared with operative treatment, nonoperative treatment decreases the potential morbidity associated with general anesthesia without worsening the infection rate.

Type I open fractures in the pediatric population differ from those in adults; in children, the fractures are bridged by a thick, vascular periosteum that facilitates fracture stability and healing. Pediatric fractures have not been as extensively studied as adult fractures, but several publications [2, 3, 10–12] have provided insight into the generally good prognosis in children. Luhmann et al. [10] reported on 65 pediatric patients with open forearm fractures treated operatively. Of the 65 fractures, 52 were type I, and 47 (90 %) of those had excellent to good results. The authors reported only one infection; it occurred in a 12-year-old with a type II open fracture. They did not find any statistically significant association between infection and fracture type or infection and time to surgical debridement. Yang and Eisler [12] studied 91 patients, 13 of whom were children, with type I fractures treated without operative irrigation and debridement. Those authors reported a 0 % infection rate with their nonoperative treatment of type I open fractures; however, 32 patients were later taken to the operating room for definitive treatment of their fractures; they did not specify how many of these patients were children. Iobst et al. [3] performed a retrospective review of 40 pediatric patients with type I open fractures in a variety of anatomic locations who were treated nonoperatively. None of the open wounds were closed primarily, and all were washed out with a povidone-iodine-saline solution at the bedside. In contrast to our study, all of their patients were admitted for intravenous antibiotics, whereas all of our patients were discharged home from the ED. The authors reported a 2.5 % infection rate. Doak and Ferrick [2] questioned whether patients with type I open fractures that were treated nonoperatively required admission for antibiotics. Their retrospective study consisted of 25 pediatric patients with type I open fractures who were discharged from the ED immediately or after 24 h of observation. They reported one case of a wound infection, and there was no delayed union or nonunion in any of their patients [2]. Patients spent an average of 7.7 ± 3.8 h in the ED during their workup and nonoperative management, a considerably shorter time than the 24 h spent by patients receiving intravenous antibiotics in other institutions. It is the senior author’s opinion that admitting the patient overnight simply to receive 24 h of antibiotics likely provides no benefit compared with receiving IV antibiotics in the ED and being discharged with oral antibiotics. In an evidence-based review, Pace et al. [11] compiled the data from the studies of Doak and Ferrick [2] and Iobst et al. [3] to make a level III recommendation for the nonoperative treatment of pediatric type I open fractures. However, they conceded that an eventual prospective level I or II study with sufficient power is still needed to make a solid recommendation for nonoperative treatment.

The decision to treat or not to treat a type I open fracture nonoperatively should still be based upon the clinical judgment of the pediatric orthopedic surgeon. Although our study shows that in most cases nonoperative treatment of type I open fractures of the forearm or tibia should be attempted, these results may not be generalizable to all patients. Operative treatment may be deemed necessary if there is worry about a concomitant compartment syndrome after high-energy trauma; if there is gross contamination after a farm accident involving dirt, pesticides, or animal feces; or if the underlying fracture pattern requires internal fixation.

Based on further literature review, another area where a level I or II study is needed is the topic of choice and duration of antibiotics for pediatric open fractures. In our study, 90 % of our patients received a dose of intravenous antibiotics in the ED, but only 70 % of the patients were discharged home with oral antibiotics, most commonly cephalexin. Although most of those patients were prescribed a 7-day course of antibiotics, there was a considerable variability, with duration ranging from 3–14 days. Lavelle et al. [13] conducted a web-based survey of academic orthopedic residency programs with regard to the treatment practices for pediatric open fractures. They found that 68 (97 %) of the 70 programs treated pediatric type I open fractures with a cephalosporin alone and that 87 % treated them with intravenous antibiotics for ≤48 h. Wound closure was also evaluated in this survey, and 90 % of programs closed the wounds in pediatric patients with type I open fractures. This finding is in contrast to our study where no patients had their wounds closed and there were no reported infections.

The major weakness of our study was the limited number of patients. The rate of infection after operative treatment of type I open fractures has been reported in the literature to be about 1.9 % [3]. To detect a 1 % increase in the rate of infection with nonoperative treatment, with a power of 0.8 and one-sided alpha of 0.05, the ideal study would need to enroll 3,210 patients in each arm. To detect a 2 % increase in the rate of infection with nonoperative treatment, the ideal study would need to enroll 997 patients in each arm. Thus, the number of patients required to enroll in randomized trials for level I evidence is quite large and will require concerted multi-institutional effort. If we pool the data from the Iobst et al. [3] and Doak and Ferrick [2] studies and combine it with our results, we find that of the 105 pediatric patients with type I open fractures treated nonoperatively among the three studies, the infection rate was 1.9 %, which is identical to that reported in the literature. In addition, there is always the possibility that patients may have experienced infections that were not documented and that the parents did not recall when they were contacted. Given the overall satisfaction of the patients at final follow-up, the presence of any latent infections was likely quite small.

In summary, nonoperative treatment of pediatric type I open fractures with subsequent discharge home from the ED appears to be safe; however, additional prospective, randomized clinical trials are needed to make a definitive level I recommendation regarding nonoperative management.
